# Measurement of hydrogen peroxide vapor in powders with potassium titanium oxide oxalate loaded cellulose pellets as probes

**DOI:** 10.1016/j.mex.2021.101405

**Published:** 2021-06-01

**Authors:** Maria H. Kastvig, Johan P. Bøtker, Ge Ge, Mogens L. Andersen

**Affiliations:** a Department of Food Science, University of Copenhagen, Frederiksberg, Denmark; b Department of Pharmacy, University of Copenhagen, Copenhagen, Denmark

**Keywords:** Hydrogen peroxide vapor, Image analysis, Powder

## Abstract

An image-based method for determining H_2_O_2_ vapor pressures in powder systems was developed based on cellulose pellets loaded with potassium titanium oxide oxalate (PTO Pellets) as probe particles. Solid titanyl salts change color after exposure to hydrogen peroxide vapor and the quantitative response of PTO pellets to H_2_O_2_ has been established by comparing reactions with H_2_O_2_ in liquid and solid states. Analysis of pictures of the color changes of PTO Pellets mixed into powders can be used to monitor the presence of ppm levels of H_2_O_2_ vapor inside powder systems such as bleach containing dry detergent powders.

• H_2_O_2_ vapor quantification in dry systems with titanyl loaded cellulose particles.
• Simple fabrication of H_2_O_2_ probe particles.
• High sensitivity with LOD 0.190 ppm H_2_O_2__._

## Graphical abstract

**Figure:**
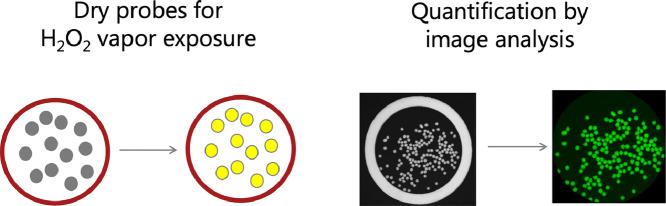

Specifications table

**Table:** 

Subject Area:	Materials Science
More specific subject area:	H_2_O_2_ vapor quantification in powder
Method name:	H_2_O_2_ vapor measurement in powders by image analysis of PTO Pellets probes
Name and reference of original method:	Paper-Based Vapor Detection of Hydrogen Peroxide: Colorimetric Sensing with Tunable Interface. [1]
Resource availability:	MATLAB software

## Method details

**Fig. 1:**
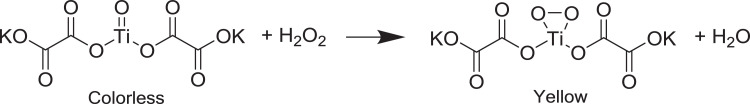
Reaction between potassium titanium oxide oxalate, PTO, and hydrogen peroxide.

Hydrogen peroxide vapor can cause stability problems of active ingredients in dry granular mixes such as powder detergents with added bleach particles [2], [3], [4], [5]. The aim of this study was to develop an experimental method to quantify the amount of H_2_O_2_ vapor inside powders at an interparticle level. Xu et al. showed that hydrogen peroxide vapor pressures could be measured by reflectance measurements of color changes of adsorbed titanyl salts absorbed into dry paper tissues [1]. Titanyl salts react with hydrogen peroxide and form yellow-colored epoxides (Fig. 1) [6]. We here report a method based on cellulose particles loaded with titanyl salt to measure H_2_O_2_ vapor in powder systems. Cellulose pellets are available commercially as Cellet®, which are microcrystalline cellulose particles. They are spherical and have open porous structures and come with diameters ranging from 100 to 1400 µm, which makes them useful as probes in many powder systems, as they can be chosen to have sizes that are within the particle size distribution of such systems. We have used the method to measure hydrogen peroxide vapor in bleach-containing detergent powder in order to estimate shelf life stability of the detergent powder in different storage conditions [7]. Cellulose pellets are not water soluble, but efficiently ad/absorb water and water soluble compounds [8].

In this work cellulose pellets were loaded with potassium titanium oxide oxalate (PTO), and the hydrogen peroxide induced color change of these PTO loaded pellets were tested as probe particles in dry systems. Xu et al. [1] developed a method based on ammonium titanyl oxalate (ATO) adsorbed into paper tissue to measure hydrogen peroxide vapor pressures. Other titanyl salts were tested, but rejected due to the higher solubility of ATO in water. Initially, we tested the two titanyl salts, ATO and PTO. Both are colorless (white) before reaction with hydrogen peroxide, and they react solely with hydrogen peroxide to form the yellow Ti(IV)-peroxide bond, while they do not give rise to color changes in the presence of a range of organic solvents (ethanol, methanol, acetone, tetrahydrofuran, hexane, toluene, ethyl acetate and chloroform) [1]. Based on the initial tests, we did not find significant differences in the properties of the two salts (supplemental material). We found that the solubility of PTO in water was adequate for our use (solutions up to 0.1 M), and PTO was finally chosen due to less safety concerns (lower toxicity) compared to ATO and a lower price.

The development of the quantitative determination of H_2_O_2_ vapor pressures were done in three steps using combinations of PTO in solution, PTO(aq), and as a solid, PTO(s), and hydrogen peroxide in solution, H_2_O_2_(aq), and as a gas H_2_O_2_(g). In Step 1 based on reactions in solutions, the exact amounts of reactants were known which allowed detailed stoichiometric and kinetic characterization of the reaction between PTO(aq) and H_2_O_2_(aq). In step 2, the results of step 1 were used to characterize the reaction between PTO(aq) and H_2_O_2_(g), which eventually in step 3 allowed development of the quantitative method based on the reaction between PTO(s) and H_2_O_2_(g). In steps 1 and 2 color measurements of titanyl salt solutions were done by UV–Vis absorbance measurements as described previously [1,^9^,10]. In step 3, PTO salt was washed out of PTO Pellets after reaction with hydrogen peroxide vapor, and the exact extent of the reaction was measured as the absorbance change of the washing solution and compared to the dry solid state color change. Further the color changes of solid PTO loaded onto cellulose pellets (PTO Pellets) could be quantified by an image-based technique, and a camera-based method for detection of hydrogen peroxide using the dry solid state of PTO was obtained, and the use of PTO Pellets to monitor H_2_O_2_ vapor was demonstrated in a powder system. The method developed is aimed for use in detergent powder where hydrogen peroxide vapor affect enzyme stability. For this reason the method needed to be easy, inexpensive and relatively fast, compared to measuring enzyme stability directly.

## Experimental details

### Materials

Potassium titanium oxide oxalate dihydrate, PTO (catalog number 14,007), ammonium titanyl oxalate monohydrate, ATO (catalog number 229989), and 30% (w/w) aqueous hydrogen peroxide solution (analytical grade) (catalog number 216763) were purchased from Sigma-Aldrich. Microcrystalline cellulose spheres (Cellets®) were purchased from Harke Pharma (Germany). Cellets® used for analysis were in the size of 100–200 µm (Pellets 100), 200–355 µm (Pellets 200), 350–500 µm (Pellets 350), 500–710 µm (Pellets 500), 700–1000 µm (Pellets 700) and 1000–1400 µm (Pellets 1000) in diameter.

### Preparation of PTO solution

PTO solutions were prepared by dissolving PTO powder in Milli-Q-water according to desired concentration (0.001 to 0.100 M) and adjusting to pH 3.0 with 3.0 M HCl solution. The PTO solutions were used for reactions with hydrogen peroxide in solution or in vapor as described below.

### Preparation of PTO pellets

PTO powder (0.0354 to 3.541 g) and 100 mL Milli-Q-water were mixed in a Blue Cap flask and adjusted to pH 3.0 with 3.0 M HCl solution. We found that 50 mL Milli-Q-water was too low an amount as the solubility of PTO was not high enough for making pellets with the desired amount of loaded PTO (17.7 wt%), when using the following steps of preparation. The PTO solution and 20.0 g pellets were added to a round-bottom flask, preferably a big flask (>0.5 L) to ensure high surface area to facilitate the evaporation. The mixture was dried on a Rotavapor (Büchi Rotavapor R-144 with a Büchi waterbath B-480 connected) and dried at 60–65 °C, 80–130 mbar and approximately 50 rpm. Rotation should not be slower, because of the risk that PTO Pellets aggregate instead of forming free-flowing single pellets. Drying under these conditions took approximately 1 h. Afterwards the flask was weighed in order to check if all water had evaporated. In case of remaining excess water, the pellet sample was placed in a heating cabinet at 60 °C overnight and weighed afterwards. Water activity was measured with an Aqualab 3 TE instrument. The possible transfer of PTO dust inside the rotavapor was tested by adding one drop of 30% hydrogen peroxide to the water in the receiver of the rotor vapor, however no yellow color formation was observed. PTO Pellets were stored in a refrigerator (5–10 °C) until further use or analysis. PTO loaded pellets had a shelf life of at least 6 months at these conditions.

### Hydrogen peroxide treatment of PTO pellets

Hydrogen peroxide solution (0.005 M to 0.90 M) was poured into four 50 mL beakers and placed in the corners of a 2.5 L airtight box (Sistema airtight plastic box). PTO Pellets (0.20 g) were placed as a monolayer in weighing boats with a bottom area ≈ 6 cm^2^ and the weighing boats were placed at the center inside the airtight box. The closed box was placed in a heating cabinet at 25 °C in the dark for a fixed time. For solution measurements, 2.5 mL PTO solution (0.01 M) was poured into semi-micro cuvettes (cat no. 634–0676) from VWR and placed in the same type of airtight boxes and under the same conditions as for PTO Pellets. Equilibrium hydrogen peroxide vapor pressures inside the boxes were calculated based on the specific concentrations of the hydrogen peroxide solutions and using the Henry's law constant *K*_h_ = (8.33 ± 0.38)·10^4^ M atm^−1^ at 25 °C [11] (for relationship between concentrations in solution and vapor pressures above solution, see supplemental material, Table S1). The precise hydrogen peroxide concentrations in solution were determined by UV–vis spectroscopy using the absorption maximum at 240 nm and the extinction coefficient ϵ =39.4 M^−1^ cm^−1^ [12,13] using a Shimadzu UV-1280 UV–Vis Spectrophotometer.

### PTO absorbance measurements

UV–Vis spectroscopic measurements of PTO were performed on a Shimadzu UV-1280 UV–Vis Spectrophotometer with semi-micro cuvettes (cat no. 634-0676) from VWR at 378 nm. All samples were dissolved in Milli-Q-water to a concentration of 0.01 M PTO.

### Hunter color measurements

Color was measured by the Hunter method with a BYK Gardner Colorimeter 45/0 (illuminant D65, observer angle 10°) as an average of three measurements. Sample was applied into a white lid (1 cm in diameter), measurements were taken from the top, and sample was shaken between measurements. a*, b* and L* values were read from the instrument. Unreacted PTO Pellets of same concentration were used as reference samples. The Hunter method provides 3 values: L*: Brightness parameter from 0 to 100. 0 = black, 100 = white. a*: Green-red parameter from −100 (green) to +100 (red). b*: Blue-yellow parameter from −100 (blue) to +100 (yellow).

### Reflectance measurements

Reflectance spectroscopy measurements were performed on a Cintra 40 and Cary 100 Bio in a flat quartz cuvette. Pellets were applied in a thin layer, and measurements were performed as scans over a range of 350 – 700 nm.

### Imaging‐based measurements

Imaging measurements were performed using VideometerLab 2 (Videometer A/S, Herlev, Denmark), the images were recorded at 8 bit depth (256 levels). For method development and determination of lower limit of detection, 0.20 g of 17.7 wt% PTO Pellets were poured into a white rubber ring (inner diameter 3.3 cm, outer diameter 4.2 cm, height ca. 0.2 cm), and images were taken with VideometerLab 2 on a black background from a distance of 22 mm from the bottom. Samples were PTO Pellets that had been treated with hydrogen peroxide vapor, and control measurements were made with PTO Pellets that had been exposed only to water vapor (only pure water in beakers in the airtight boxes) under similar conditions. Greyscale images recorded at 450 nm were analyzed using the software MATLAB R2017a. The pellets were segmented using the watershed algorithm and multiple images were sorted using the sort_nat function version 1.4 developed by Douglas M. Schwarz [14]. The intensities of the individual PTO Pellets at 450 nm were transformed into an average value called mean (450).

## Method validation

### Initial testing of PTO and ATO

The reactions in aqueous solution between hydrogen peroxide and the two titanyl salts ATO and PTO were compared in order to identify optimal reaction conditions and the best probe molecule (Figure S1 – supplementary material). The absorbance changes at 378 nm generated by the reaction of ATO and PTO with H_2_O_2_ were not affected by varying the temperature (20–80 °C), and the absorbance changes were stable over time (20 min – 43 h). The changes were however dependent on the pH during the reaction, and the maximum absorbance was obtained at pH 3, while the absorbances decreased considerably at pH values above 5. At pH 7.0 precipitates were observed in the reaction mixture. There were no differences between properties of the ATO and PTO salts, and it was decided to use PTO salt for the rest of the experiments, due to lower price and lower toxicity of this compound [15,16].

### Reaction between PTO and H_2_O_2_ in solution

**Fig. 2:**
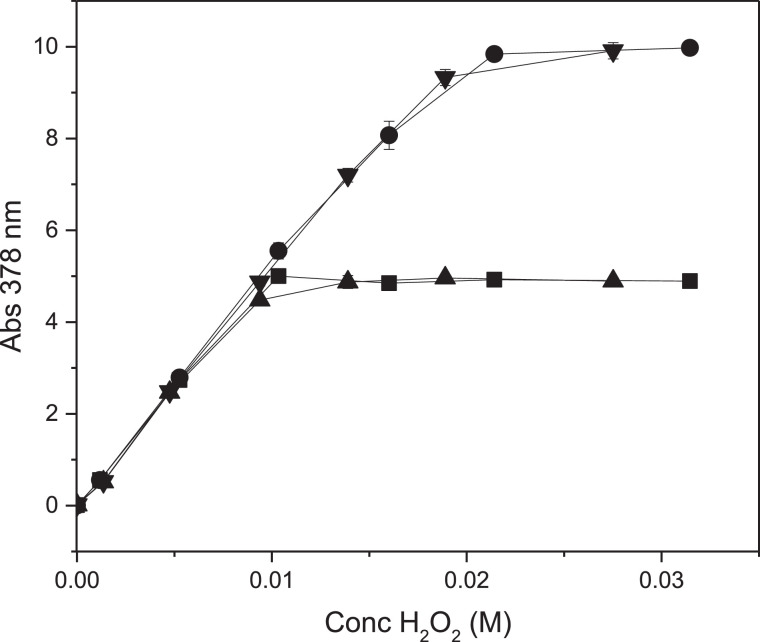
Absorbance changes during reactions between 0.01 or 0.02 M PTO(aq) (pH 3.0) and H_2_O_2_(aq) at room temperature. Concentrations of PTO: 0.01 M (■), 0.02 M (●), 0.01 M (▲), 0.02 M (▼) (mean ± st. dev., *n* = 3).

The stoichiometry of the reaction between PTO and H_2_O_2_ was examined by recording the absorbance changes generated by mixing 0.01 M or 0.02 M PTO solutions and hydrogen peroxide solutions of 0.0 - 0.032 M (Fig. 2). The absorbance increased linearly with the amount of added H_2_O_2_ and levelled off when the amount of H_2_O_2_ exceeded a 1:1 mole ratio with PTO confirming that hydrogen peroxide and PTO react on a mole basis of 1:1 [1,^9^,10]. This allowed the determination of the extinction coefficient at 378 nm of the titanyl epoxide as 487.0 ± 7.7 M^−1^ cm^−1^. Furthermore, the linearity of the data shows that standard curves for determining H_2_O_2_ solution concentrations can be constructed using PTO as probe and vice versa.

### Reaction between PTO (aq) and H_2_O_2_ (g)

**Fig. 3:**
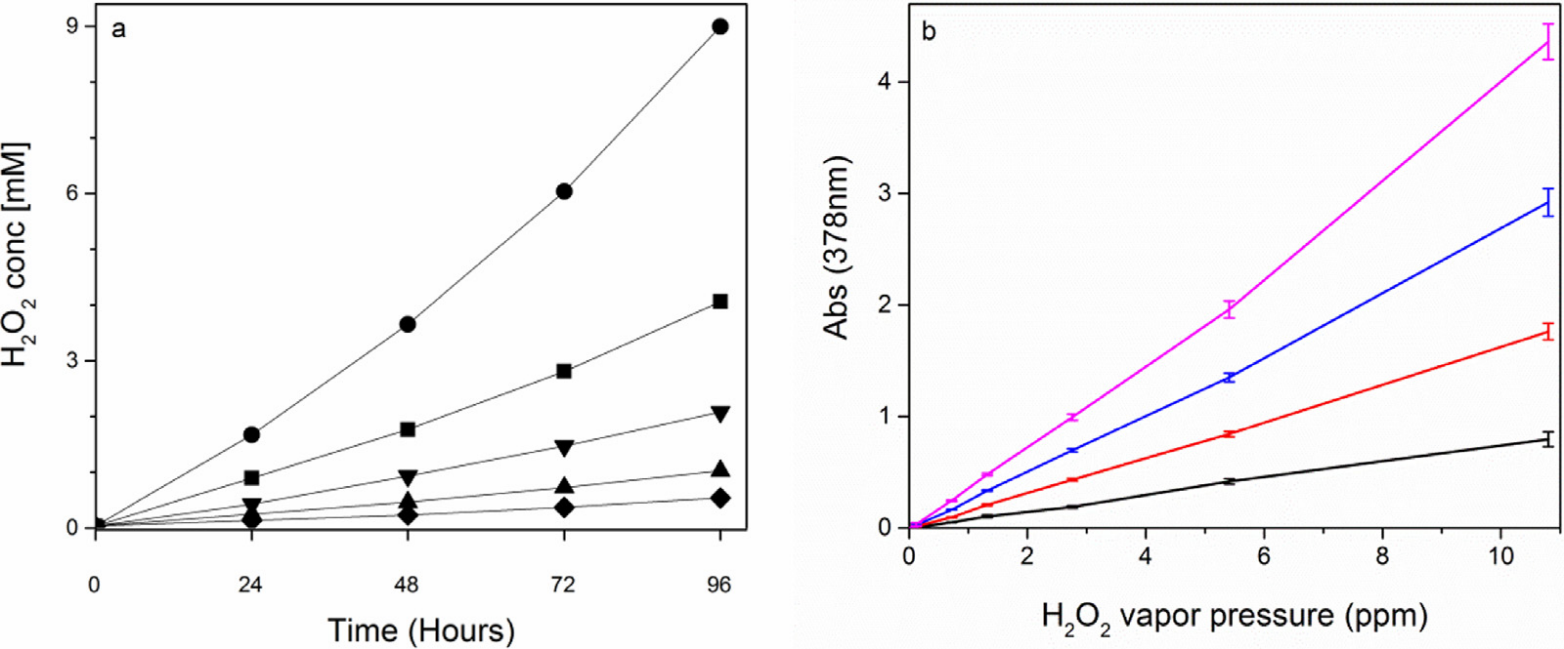
Reaction between hydrogen peroxide vapor and 0.01 M potassium titanium oxide oxalate (PTO) solution: a) Hydrogen peroxide uptake at different H_2_O_2_ vapor pressures (10.8 ppm (●), 5.4 ppm (■), 2.8 ppm (▼), 1.3 ppm (▲), 0.7 ppm (♦)) at 25 °C. b) PTO solution absorbances after 24 h (black), 48 h (red), 72 h (blue) and 96 h (purple) as a function of H_2_O_2_ vapor pressure (mean ± st.dev., *n* = 4). Linear regression gave slopes 0.074 ppm^−1^ (24 h, R^2^ = 0.999), 0.16 ppm^−1^ (48 h, R^2^ = 0.999), 0.27 ppm^−1^ (72 h, R^2^ = 0.998), and 0.39 ppm^−1^ (96 h, R^2^ = 0.996).

Samples of 2.5 mL 0.01 M PTO solutions in semi-micro cuvettes were exposed to hydrogen peroxide vapor by storing them next to hydrogen peroxide solutions inside a closed box. The hydrogen peroxide vapor pressure was varied between 6.01·10^−8^ atm and 1.08·10^−5^ atm (equivalent to 0.06 – 10.8 ppm) by using hydrogen peroxide solutions with concentrations in the range 0.005 M to 0.910 M. The amount of H_2_O_2_ taken up by the PTO solutions was calculated based on the extinction constant determined for the titanyl epoxide and the 378 nm absorbance changes. The uptake of H_2_O_2_ increased with time and with increasing hydrogen peroxide vapor pressure (Fig. 3a). At a given time point, the uptake of H_2_O_2_ determined as the absorbance increase was proportional to the hydrogen peroxide vapor pressure (Fig. 3b). Experiments also showed that the amount of H_2_O_2_ uptake only depended on the hydrogen peroxide vapor pressure and not on the concentration of the PTO solutions when they had similar surface areas (Figures S2-S3 – supplementary material). The determination of hydrogen peroxide vapor pressures can therefore be carried out using the proportionality constants and the observed color change of the PTO solutions after exposure to the atmosphere of interest during the specified time. The assay results showed that the colorimetric response is time dependent, and H_2_O_2_ uptake in PTO aqueous solution was validated during 96 h of reaction time by these results. The results clearly showed that lower H_2_O_2_ vapor concentrations can be measured after 96 h than 24 h. However, from a practical point of view it was decided to use 24 hour time point for further experiments since this would allow quicker generation of results. Furthermore, the 24 hour time point was well within the validated time (96 h). As further experiments would show, 24 h was a suitable time for the acquired hydrogen peroxide vapor concentration.

### Reaction of H_2_O_2_ with PTO pellets

Pellets with diameters in the range 500–710 μm (Pellets 500) were loaded with different concentrations of PTO (0.035 wt%–35.4 wt%) by mixing pellets into an aqueous solution of PTO. Water was then dried off in two steps: First under vacuum at 60–65 °C and afterwards by heating at 60 °C in an oven, until the weight loss corresponded to the amount of applied water. The resulting product was free-flowing white PTO Pellets that looked like the original untreated pellets, and with 17.4% ± 0.8% (*n* = 5) water activity after drying. At high concentration of PTO (35.4 wt%), solids were observed to easily break of pellets, and only PTO Pellets with lower concentrations were therefore used for further testing.

Exposing PTO Pellets to atmospheres with 5.15·10^−6^ atm H_2_O_2_ vapor pressures gave color changes that could be quantified by Hunter reflectance measurements (Figure S4 - supplementary material). There were only small differences between color measurements of pellets loaded with PTO in the range 1.8 wt% to 17.7 wt%. Pellets with the low concentration of PTO (0.035 wt%) did not give measureable color change and these pellets were not tested further. Even though 1.8–17.7 wt% PTO Pellets gave similar results, it was decided to use mainly 17.7 wt% for the next experiments, based on the assumption it would give a more stable response towards very low amounts of hydrogen peroxide.

The color formation by exposing PTO Pellets to H_2_O_2_ vapor was studied using pellets with sizes of 100, 200, 350, 500, 700 and 1000 µm (Figure S5 – supplementary material). The L*-value systematically decreased with increasing size of pellets, but since a*- and b*-values were not clearly affected by the pellet size, it was concluded that pellets of different sizes should be applicable.

**Fig. 4:**
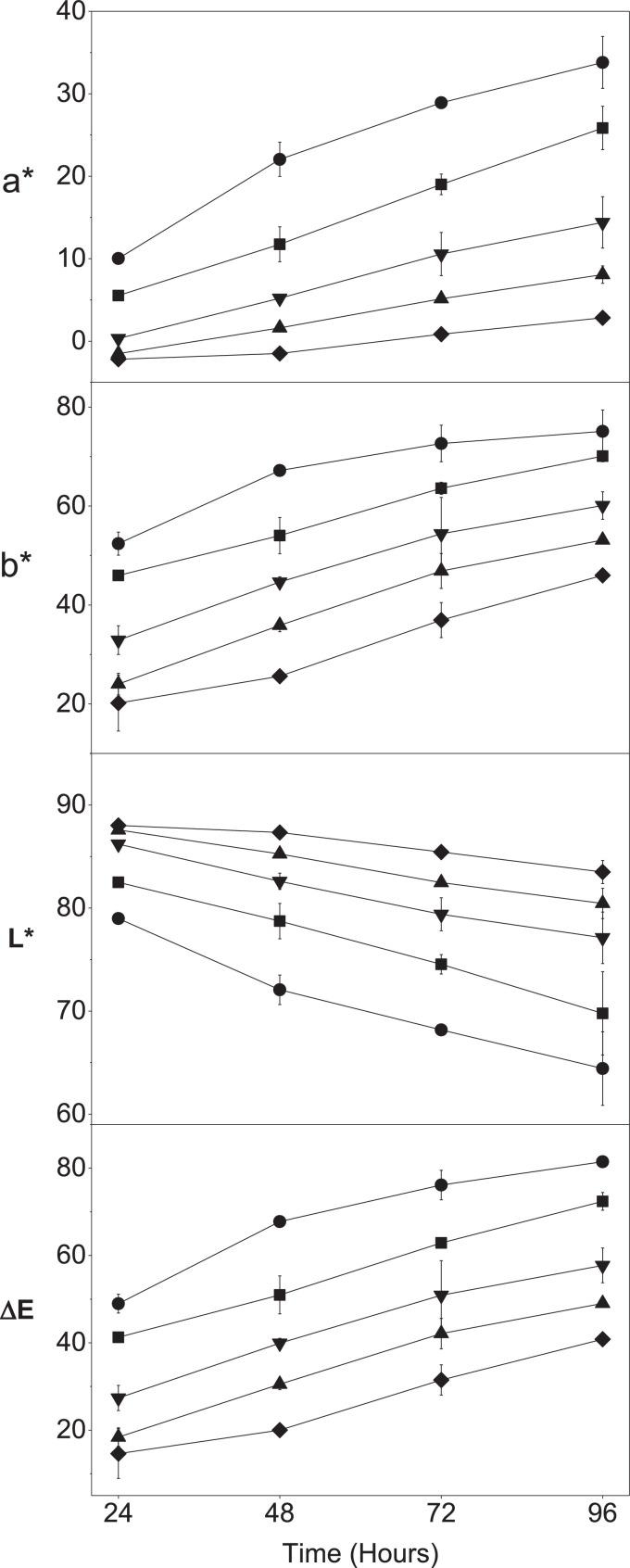
Color measurements by the Hunter method of 17.7 wt% PTO Pellets 500 after reaction with hydrogen peroxide vapor over time (24–96 h). Vapor pressures H_2_O_2_: 1.08·10^−5^ atm (●), 5.41·10^−6^ atm (■), 2.76·10^−6^ atm (▼), 1.32·10^−6^ atm (▲), 7.21·10^−7^ atm (♦). Hunter values: L*: 0 = black, 100 = white; a*: −100 = green, +100 = red; b*: −100 = blue, +100 = yellow. Mean ± st.dev., *n* = 2.

**Fig. 5:**
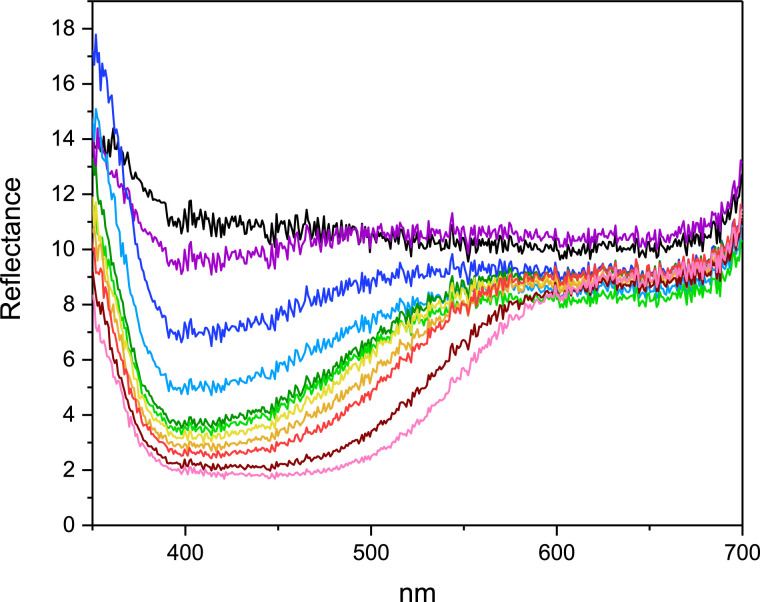
Reflectance spectra of 17.7 wt% PTO Pellets 500 after incubation in H_2_O_2_ vapor of estimated 7.21·10^−7^ atm to 1.08·10^−5^ atm for 24 and 96 h (*n* = 1). From top to bottom (~400 nm): Untreated sample (black), 7.21·10^−7^ atm for 24 h (purple), 1.32·10^−6^ atm for 24 h (dark blue), 2.76·10^−6^ atm for 24 h (light blue), 5,41·10^−6^ atm for 24 h (dark green), 7.21·10^−7^ atm for 96 h (light green), 1.32·10^−6^ atm for 96 h (yellow), 1.08·10^−5^ atm for 24 h (orange), 2.76·10^−6^ atm for 96 h (red), 5,41·10^−6^ atm for 96 h (bordeaux) and 1.08·10^−5^ atm for 96 h (pink). (For interpretation of the references to color in this figure legend, the reader is referred to the web version of this article.)

Pellets 500 with 17.7 wt% PTO were exposed to H_2_O_2_ vapor pressures from 7.20·10^−7^ atm to 1.08·10^−5^ atm between 24 and 96 h, and the color formation was measured using the Hunter method (Fig. 4) and by obtaining reflectance spectra (Fig. 5). The PTO Pellets became more yellow (increasing b*-value) during exposure to all vapor pressures (reflectance intensity at approximately 400 nm). Redness (a*-value) of the PTO Pellets increased during the exposure, especially at the high vapor pressures, changing the color from yellow towards orange. This was also seen in the reflectance spectra that broadened from approximately 400 nm towards 550 nm. Whiteness (L*-value) of PTO Pellets decreased in all pellets over time, with the highest decrease at the highest H_2_O_2_ vapor pressure. These color differences were also clearly visible to the naked eye (Figure S6 – supplementary material).

### Comparing reactions of hydrogen peroxide with PTO (aq) and PTO (s)

**Fig. 6:**
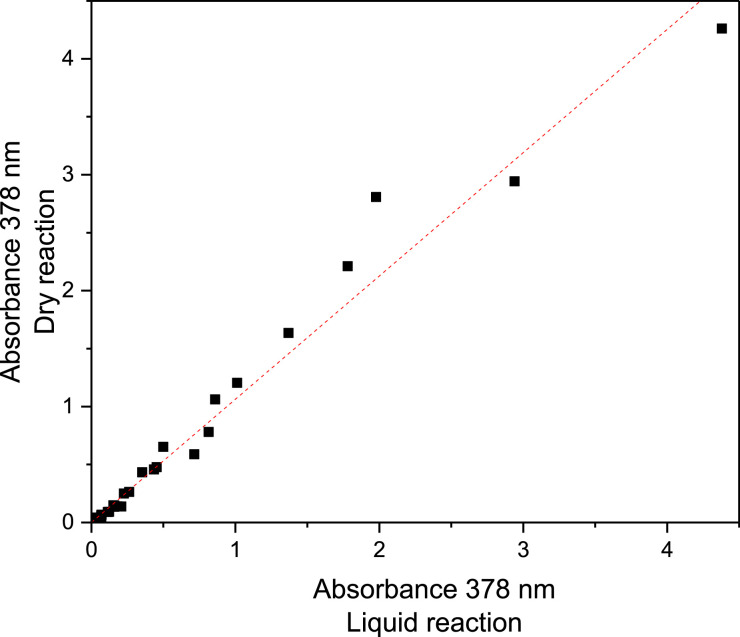
Absorbance at 378 nm of 2.5 mL 0.01 M PTO solution after reaction with H_2_O_2_ vapor with vapor pressures 6.01·10^−8^–1.08·10^−5^ atm for 24–96 h in either 2.5 mL semi-macro cuvettes (*x*-axis) or as PTO Pellets 500 or 1000, after extracting the PTO salt from the pellets (to a final concentration of 0.01 M in the extracted PTO solution). Dotted line represents best fit, *y* = 1.0635x (R^2^=0.9753).

The amount of PTO in the pellets that had reacted with H_2_O_2_ was evaluated by comparing the absorbance changes (378 nm) of 0.01 M PTO solutions and of extracted PTO from PTO Pellets that had been exposed to the same H_2_O_2_ vapor pressures under the same conditions. The PTO was extracted from the pellets with Milli-Q water (0.20 g PTO Pellets to 0.01 L H_2_O) to reach the concentration 0.01 M PTO which allowed the direct comparison of the absorbance at 378 nm. Data for reaction with H_2_O_2_ vapor up to vapor pressures 1.08·10^−5^ atm for 96 h were included in this comparison (Fig. 6). This gave a linear relationship between the amounts of PTO that had reacted in solution and in the solid state with slope of 1.06 (R^2^ = 0.9753), which confirmed that the reactivity and the response of PTO towards H_2_O_2_ was the same in solution and in PTO Pellets. Data was also compared between absorbance changes of extracted PTO solutions from PTO Pellets 500 and 1000, which gave a linear relationship with slope of 1.01 (R^2^ = 0.9749) indicating a similar response of the two types of pellets (Table S2 and Figure S7 – Supplementary Material).

### Image based quantification of H_2_O_2_ with PTO pellets

**Fig. 7:**
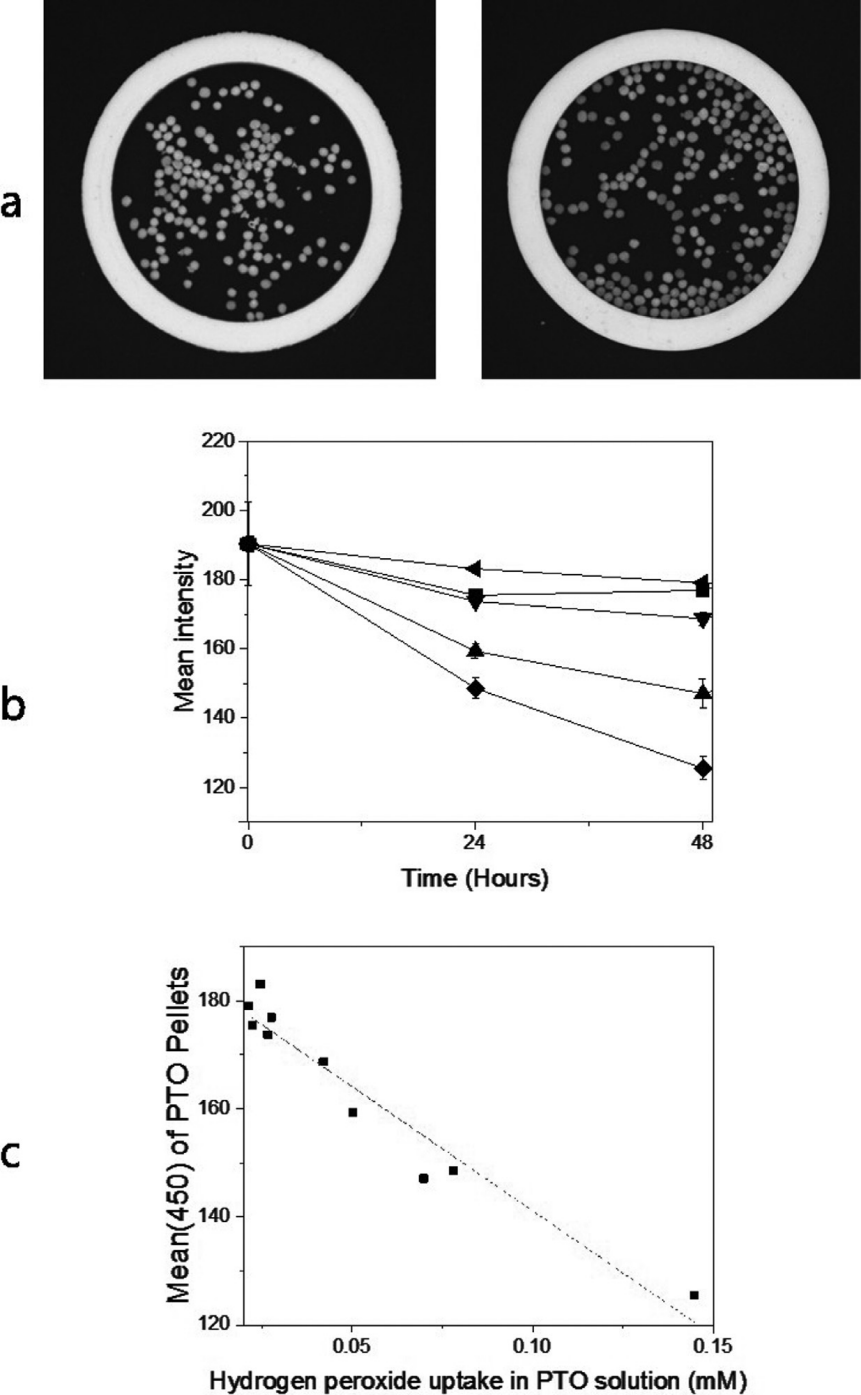
Videometer data of reference samples (17.7wt% PTO Pellets 1000). a. Example of greyscale pictures at 450 nm before reaction with H_2_O_2_ vapor (left) and after incubation in H_2_O_2_ vapor (0.7 ppm) for 96 h (right). b: Mean intensity from greyscale image at 450 nm after incubation in H_2_O_2_ vapor conditions at 25 °C (mean ± st.dev., *n* = 2). Vapor pressures H_2_O_2_: 0.00 atm (◄), 6.08·10–^8^ atm (■) 1.20·10^−7^ atm (▼), 3.60·10^−7^ atm (▲), 7.21·10^−7^ atm (♦). c: Comparison of Videometer mean results (mean(450)) to H_2_O_2_ uptake concentration (calculated from absorbance at 378 nm in solution PTO (0.01 M) stored in same conditions as PTO Pellets for 24 and 48 h). Samples were 17.7 wt% Pellets 1000 and 500 reacted with H_2_O_2_ vapor pressures from 6.01·10^−8^ atm to 7.21 ·10^−7^ atm over time (0–48 h) at 25 °C.

Greyscale images recorded at 450 nm were obtained of 17.7 wt% PTO Pellets 1000 and 500 that had been exposed to H_2_O_2_ vapor pressures between 6.01·10^−8^ atm and 7.21·10^−7^ atm at 25 °C (Fig. 7a). The mean color intensities (mean(450)) of the PTO Pellets, which were obtained using a MATLAB image analysis script, decreased with exposure time and H_2_O_2_ vapor pressure (Fig. 7b). PTO in solution (0.01 M) was stored in the same airtight boxes as the PTO Pellets, and by using the titanyl peroxide extinction coefficient, the hydrogen peroxide vapor taken up by the solution were calculated from the absorbances. A plot of the mean intensities of PTO Pellet colors determined by the image analysis against the uptake of hydrogen peroxide in PTO solutions gave a linear fit, showing that the PTO Pellet image data can be used to quantify the exposure to H_2_O_2_ vapors (Fig. 7c). Since the hydrogen peroxide uptake by PTO at a given exposure time is proportional to the H_2_O_2_ vapor pressure, the image analysis data can be used directly calculate the H_2_O_2_ vapor pressure. Based on data from experiments with 24 h exposure at 25 °C of 17.7 wt% PTO Pellets 1000 in water vapor (blanks, *n* = 7) and in an atmosphere with H_2_O_2_ vapor pressure (3.60·10^−7^ atm) (samples, *n* = 7), the lower limit of detection (LOD) was calculated as corresponding to a H_2_O_2_ vapor pressure from solution of 0.016 M H_2_O_2_, corresponding to a vapor pressure of 1.90·10^−7^ atm (ca. 0.19 ppm). Determination of LOD was performed accordingly to method described in [17]. The signal detection limit (SDL) was calculated for the imaging method after 24 h by comparison between blanks (*n* = 7) and samples (*n* = 7), as the mean value of blanks + 3 standard deviations of samples. The mean of the blanks was 191.2, sample standard deviation was 2.99 and SDL was calculated to 182.3. Mean of samples subtracted with the mean of blanks was proportional to sample concentration (Eq. (1)), and a constant-value, m, was deduced from this relationship. The value of m was calculated to be 568.49 mean intensity of PTO Pellets·M^−1^.

(1) $${\mathrm{Mean}}_{samples}-{\mathrm{Mean}}_{blanks}=m\cdot {\mathrm{Conc}}_{samples}$$

Lower limit of detection (LOD) was calculated as 3 sample standard deviations divided with m-value.

SDL in solution at absorbance at 378 nm was also determined and gave an absorbance value of 0.0093. However, sample mean was absorbance of 0.0076, and thereby below SDL. The conclusion was therefore that the imaging technique gave a lower LOD-value than absorbance measurements in 0.01 M solution.

### Application of PTO pellets in powder

The reason for developing the image-based method was to measure interparticle hydrogen peroxide vapor in powder systems. Images of PTO Pellets in powder systems should be consistent to the images of PTO Pellets outside a powder system, and the samples were therefore analyzed inside a circle with a dark background, to have a reproducible and constant data analysis area.

**Fig. 8:**
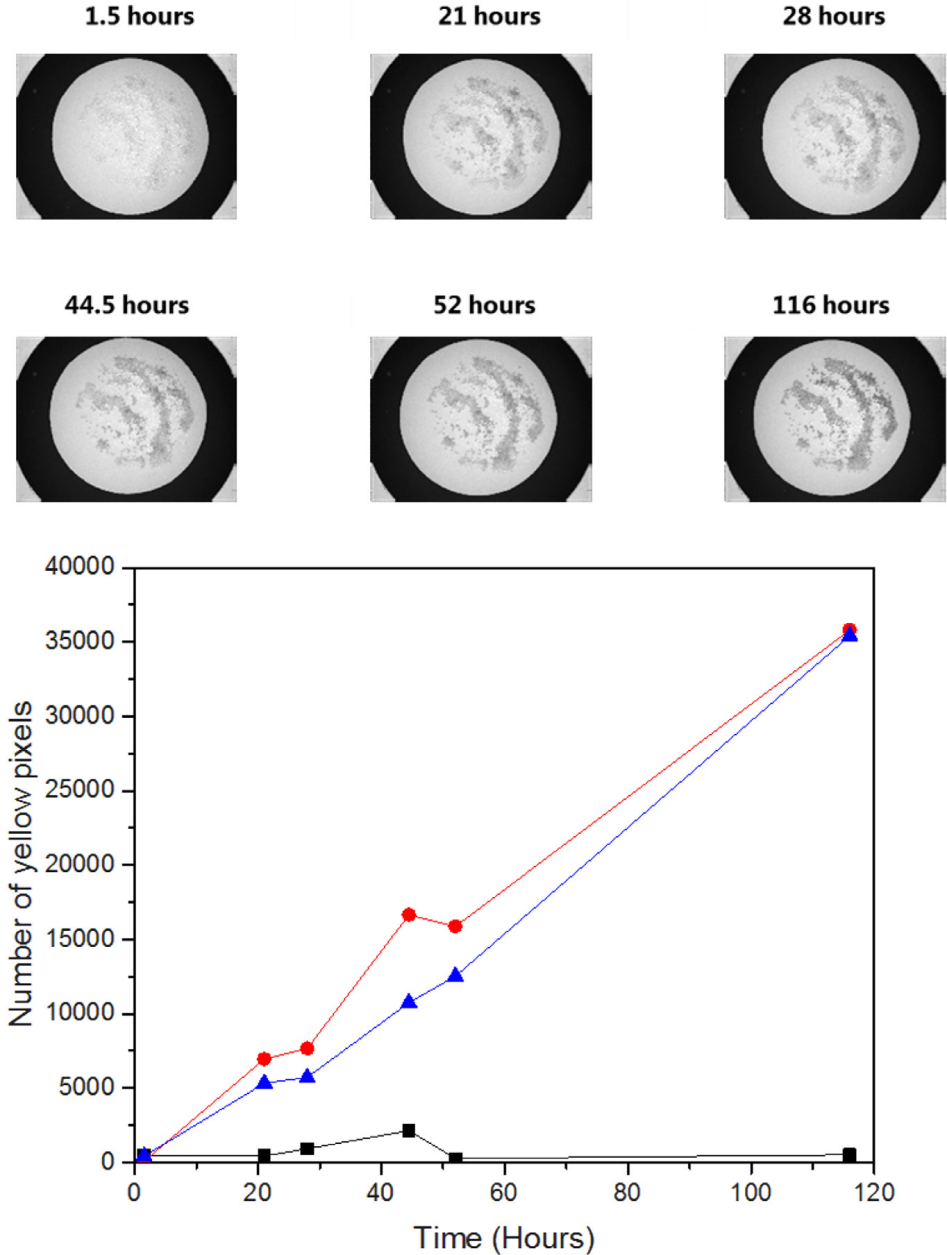
Yellow color development determined by image analysis of 3.5 wt% PTO Pellets 500 glued to cardboard circles stored in two different commercial detergent powders. Detergent 1 (Red circles). Detergent 2 (Blue triangles). Control, PTO Pellets stored without detergent (Black squares). Images shown on top are greyscale images at 450 nm of samples stored in Detergent 2 with 15–30% oxygen based bleaching agents.

Initially it was tested to fixed PTO Pellets by gluing the pellets onto blue cardboard circles and then storing the pellets-loaded cardboard pieces in detergent powder while shaking the samples. Retaining the pellets in the same positions over time would keep variation between images to a minimum. The cardboard pieces were taken out over time (0–116 h) and images were recorded at 450 nm, before the cardboard pieces where inserted into detergent powder again. Two different commercial detergent powders labeled either “5–15% oxygen based bleaching agents” (Detergent 1) or “15–30% oxygen based bleaching agents” (Detergent 2) were used. PTO Pellets (0.25 g, 500 µm diam.) with 3.5 wt% PTO were glued to paper circles (7.0 cm diam.) and placed in a petri dish of 8.8 cm with 0.5 g detergent powder. The samples were kept closed and shaken continuously at room temperature. The control sample of PTO pellets was kept in a petri dish without detergent powder. The image analysis showed increasing yellow color of the PTO pellets with time demonstrating the presence of H_2_O_2_ vapor in both detergents, whereas no color changes were observed for the samples stored without detergent (Fig. 8). The similar response of the PTO Pellets in the two detergents shows that the samples have similar H_2_O_2_ vapor pressures. Disturbance from powder covering the PTO Pellets was small, and it was possible to physically separate the pellets from detergent powder. However, this method did not apply model particles into a powder system, since the pellets were not free-flowing and therefore, this method was not further developed. It was decided to skip the shaking as well, in order to simulate real storage conditions of a powder.

**Fig. 9:**
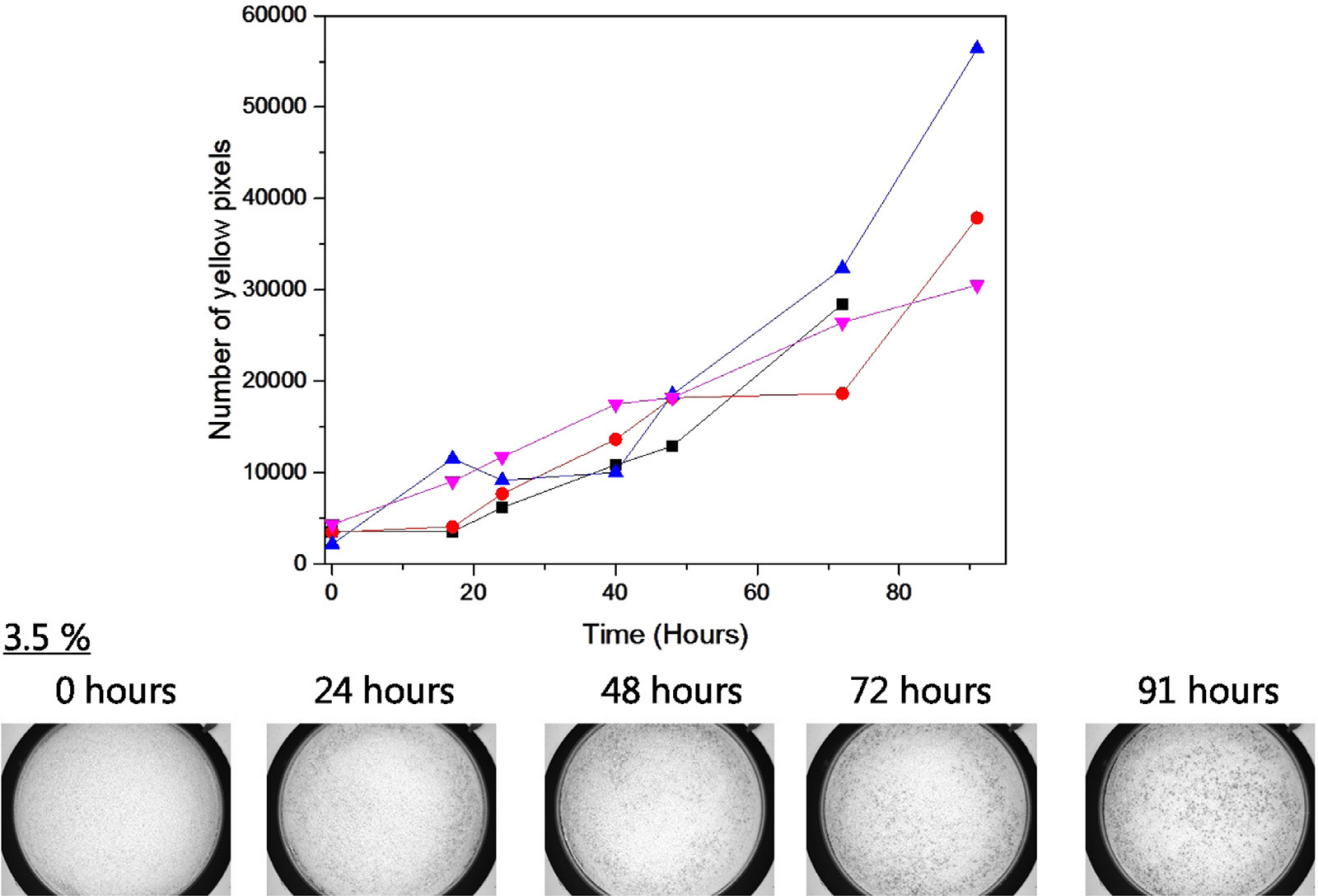
Analysis of different loadings of PTO Pellets in detergent powder. Mixtures of 5.0 g detergent powder and 0.75 g PTO Pellets in petri dish with diameter 8.8 cm stored at 25 °C. Images were taken at 450 nm and color development was transformed into a mean value. Pellets were loaded with PTO in concentration of 0.2 wt% (■), 0.4 wt% (●), 3.5 wt% (▲) and 17.7 wt% (▼). Examples of images of 3.5 wt% samples are shown.

The effect of the PTO loading in the pellets on the sensitivity towards H_2_O_2_ vapor was tested with a commercially available detergent powder without enzyme. Detergent powder (5.0 g) and PTO Pellets (0.75 g) were mixed and transferred to petri dishes (diam. 8.8 cm), and the sample only just covered the bottom of the dish. The pellets were loaded with 0.2 wt%, 0.4 wt%, 3.5 wt% and 17.7 wt%. The closed dishes were stored in the dark at 25 °C and Videometer images were recorded at regular intervals. Analysis of the images showed that all PTO Pellets gave the same linear color increase over time, and that the PTO loading was not the limiting the response and sensitivity towards H_2_O_2_ vapor of the pellets. (Fig. 9).

In summary, we have succeeded in producing model particles – PTO Pellets – that can quantify H_2_O_2_ vapor in powder systems. Color change in solid state correlated to the absorbance change of PTO solution at 378 nm after exposure to hydrogen peroxide vapor. The color change of the pellets can be monitored by image analysis of the pellets over time. Thus, the accumulated vapor concentration of hydrogen peroxide over time can be monitored using the image-based analysis of pellets. The method can be used for monitoring of powdered enzyme stability in detergent powder, in which hydrogen peroxide vapor is believed to be the main reason for enzyme destabilization over time. The PTO Pellets can be added to the enzyme-containing detergent powder, and the color change of PTO Pellets is followed by image-analysis of the powder mixture over time. This will be an easy approach to use for predicting stability of enzymes in new detergent formulations.

## Declaration of Competing Interest

The authors declare that they have no known competing financial interests or personal relationships that could have appeared to influence the work reported in this paper.

## Appendix Supplementary materials

Image, application 1
