# Supplementary material for: Measurement of hydrogen peroxide vapor in powders with potassium titanium oxide oxalate loaded cellulose pellets as probes
Source: MethodsX. 2021 Jun 1;8:101405. doi: 10.1016/j.mex.2021.101405 (PMC8374476; doi:10.1016/j.mex.2021.101405)
Supplement: Supplementary file 1 [file mmc1.docx]

# Supplementary material to

# Measurement of Hydrogen Peroxide Vapor in Powders with Potassium Titanium Oxide Oxalate Loaded Cellulose Pellets as Probes

# Maria H. Kastvig, Johan P. Bøtker, Ge Ge, Mogens L. Andersen. University of Copenhagen.

**Table S1**. Equilibrium H_2_O_2_ vapor pressures above aqueous H_2_O_2_ solutions. Calculated based on Henry’s law constant (*K*_H_ = (8.33 ± 0.38)·10^4^ M·atm^-1^ at 25 °C) [1].

| - **Solution** - **H_2_O_2_ concentration (M)** | - 0.005 | - 0.010 | - 0.030 | - 0.060 | - 0.110 | - 0.230 | - 0.450 | - 0.900 |
| --- | --- | --- | --- | --- | --- | --- | --- | --- |
| - **Equilibrium H_2_O_2_ vapor pressure (atm)** | - 6.01∙10^-8^ | 1.20∙10^-7^ | - 3.60∙10^-7^ | - 7.21∙10^-7^ | - 1.32∙10^-6^ | - 2.76∙10^-6^ | - 5.41∙10^-6^ | - 1.08∙10^-5^ |

#### References

[1] D.W. OSullivan, M.Y. Lee, B.C. Noone, B.G. Heikes, Henry's law constant determinations for hydrogen peroxide, methyl hydroperoxide, hydroxymethyl hydroperoxide, ethyl hydroperoxide, and peroxyacetic acid, J Phys Chem-Us, 100 (1996) 3241-3247.

**Table S2**. Data for absorbance in aqueous PTO solution (0.01 M) and color and image analysis results for 17.7 wt% PTO Pellets 1000 incubated in 6.01∙10^-8^ atm to 7.21∙10^-7^ atm for 0 to 96 hours. Absorbance in solution refers to absorbance at 378 nm of 0.01 M aqueous solution of PTO after incubation in hydrogen peroxide vapor, wash-off absorbance refers to absorbance at 378 nm of washed out PTO salt corresponding to 0.01 M from PTO Pellets after incubation in hydrogen peroxide vapor. a*, b* and L* refers to the Hunter measurements of PTO Pellets, mean (n=2).

| Incubaion time | Equilibrium  pressure of H_2_O_2_ (atm) | 0.01 M PTO solution | | | PTO Pellets | | | | |
| --- | --- | --- | --- | --- | --- | --- | --- | --- | --- |
|  |  | 378 nm Abs | | Calculated uptake of H_2_O_2_ (M) | a* | b* | L* | Videometer mean | 378 nm Abs Wash off |
| 24 hours | 7.21∙10^-7^ | 0.038 | 0.078 | | 0.83 | 27.80 | 65.56 | 148.6 | 0.028 |
|  | 3.60∙10^-7^ | 0.025 | 0.050 | | 1.41 | 21.80 | 65.60 | 159.3 | 0.015 |
|  | 1.20∙10^-7^ | 0.013 | 0.027 | | 0.72 | 16.64 | 66.65 | 173.8 | 0.006 |
|  | 6.01∙10^-8^ | 0.011 | 0.023 | | 0.46 | 15.94 | 65.71 | 175.6 | 0.003 |
|  | 0.00 | 0.012 | 0.025 | | -0.08 | 16.89 | 63.92 | 183.1 | 0.004 |
| 48 hours | 7.21∙10^-7^ | 0.071 | 0.145 | | 0.18 | 34.74 | 61.67 | 125.5 | 0.068 |
|  | 3.60∙10^-7^ | 0.034 | 0.070 | | -0.44 | 29.33 | 63.39 | 147.1 | 0.033 |
|  | 1.20∙10^-7^ | 0.021 | 0.042 | | -1.37 | 19.21 | 65.57 | 168.7 | 0.011 |
|  | 6.01∙10^-8^ | 0.014 | 0.028 | | -0.52 | 16.27 | 64.63 | 176.9 | 0.004 |
|  | 0.00 | 0.011 | 0.022 | | -0.49 | 15.99 | 66.66 | 179.1 | 0.004 |
| 72 hours | 7.21∙10^-7^ | 0.153 | 0.314 | | -0.13 | 30.82 | 66.35 | 136.9 | 0.094 |
|  | 3.60∙10^-7^ | 0.060 | 0.123 | | -1.58 | 24.09 | 67.99 | 163.6 | 0.038 |
|  | 1.20∙10^-7^ | 0.025 | 0.051 | | -1.05 | 16.62 | 67.77 | 185.6 | 0.015 |
|  | 6.01∙10^-8^ | 0.018 | 0.036 | | -1.04 | 14.24 | 67.89 | 196.4 | 0.008 |
|  | 0.00 | 0.013 | 0.027 | | -0.14 | 16.91 | 65.59 | 176.7 | 0.004 |
| 96 hours | 7.21∙10^-7^ | 0.159 | 0.325 | | 2.53 | 32.51 | 63.62 | 128.4 | 0.146 |
|  | 3.60∙10^-7^ | 0.069 | 0.142 | | -0.85 | 25.79 | 65.33 | 154.5 | 0.053 |
|  | 1.20∙10^-7^ | 0.029 | 0.060 | | -1.40 | 17.50 | 66.95 | 186.6 | 0.020 |
|  | 6.01∙10^-8^ | 0.018 | 0.037 | | -0.58 | 14.54 | 67.25 | 195.0 | 0.011 |
|  | 0.00 | 0.013 | 0.027 | | -0.14 | 16.91 | 65.59 | 177.0 | 0.004 |

**Figure S1.** Absorbance changes at 378 nm during reactions of H_2_O_2_ (0.005 M) with 0.01 M potassium titanium oxide oxalate (●) and ammonium titanyl oxalate (■). Solutions were diluted 4 times before measurement. a: Absorbance at pH ~3.5 at different temperatures (20-80 °C). b: Absorbance at room temperature (~ 25 °C) in reactions with pH from 1.0 to 7.0. c: Time dependence of absorbance at room temperature without pH adjustment. d: Absorbance measurements at pH 3.0, room temperature from 0 to 2580 minutes after reaction.

**Figure S2**. Absorbance at 378 nm of 2.5 mL potassium titanium oxide oxalate (PTO) solution in semi-micro cuvettes reacting with hydrogen peroxide vapor at 25 °C. 0.02 M PTO stored over a 24% H_2_O_2_ solution: (■). 0.02 M PTO stored over a 12% H_2_O_2_ solution: (▲).0.01 M PTO stored over a 12% H_2_O_2_ solution: (●) (mean ± st.dev., n=2).


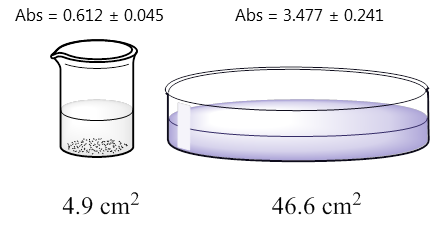


**Figure S3.** 5.0 mL 0.01 M solution of potassium titanium oxide oxalate (PTO) was poured into either a glass beaker with surface area of 4.9 cm^2^ or a petri dish with surface area of 46.6 cm^2^. Each container was placed in a ¾ L airtight glass-container. The bottom of the container was covered with 10 mL 0.275 M hydrogen peroxide solution. The beakers were raised from the bottom by placing them on plastic holders for Eppendorf tubes, and the containers were incubated for 24 hours at 25 °C. Afterwards absorbance at 378 nm was measured of PTO solution (mean ± st.dev., n=3).

**Figure S4**. Color changes of Pellets 500 with PTO concentrations from 0.18 wt% - 17.7 wt% during storage at 25 °C over a 0.4289 M hydrogen peroxide solution, providing a hydrogen peroxide vapor pressure of 5.15∙10^-6^ atm. 0.18 wt% PTO: (■), 0.4 wt% PTO: (●), 1.8 wt% PTO (▲), 3.5 wt% PTO (▼), 17.7 wt% PTO(♦). Hunter measurements (a*, b*, L*-values) of PTO Pellets and absorbance values at 378 nm (after multiplication with dilution factor) of PTO Pellets in Milli-Q-water corresponding to a concentration of 0.01 M PTO (mean ± st.dev., n=2).

**Figure S5.** Color measurements of 17.7 wt% PTO Pellets 100-1000 after reaction for 48 or 72 hours at 25 °C with hydrogen peroxide vapor produced from 0.103 M hydrogen peroxide solution, corresponding to a vapor pressure of 1.24∙10^-6^ atm. 48 hours: (■), 72 hours: (○) (mean ± st.dev., n=2).


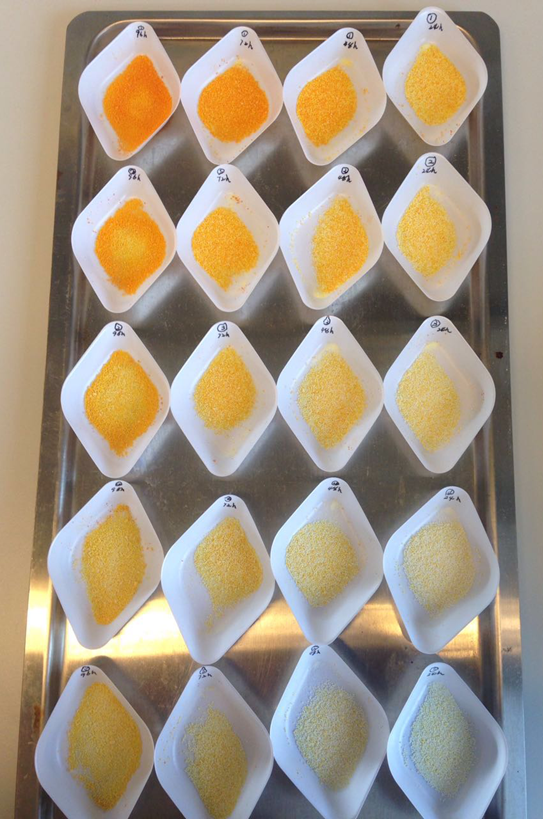


**Figure S6.** PTO Pellets 500 (17,7 wt%)) after incubation in H_2_O_2_ vapor. Incubation time from left to right: 96 hours, 72 hours, 48 hours and 24 hours. Vapor pressure of H_2_O_2_ from top to bottom: 1.08∙10^-5^ atm, 5.41∙10^-6^ atm, 2.76∙10^-6^ atm, 1.32∙10^-6^ atm and 7.21∙10^-7^ atm.

**Figure S7.** Absorbance at 378 nm of 0.01 M PTO solution after exposure to H_2_O_2_ vapor (6.01∙10^-8^ - 7.21∙10^-7^ atm) for 24-96 hours as PTO Pellets 500 (x-axis) or 1000 (y-axis), after washing off the PTO salt. Dotted line represents best fit, y = 1.0135x (R^2^=0.9747).

#### Development of data script

A data script was developed for the image analysis. The final script measured intensity in a single pixel in each pellet, but in initial testing we had tried to use the entire area in each pellet. This data treatment was tested on images of 17.7 wt% PTO Pellets 1000, reacted with H_2_O_2_ vapor of 1.20∙10^-7^ atm for 0-96 hours (n=7) at room temperature. 0.20 g pellets were poured into a white ring (inner diameter 3.3 cm, outer diameter 4.2 cm), and images were taken with Videometer 2 on a black background from a distance of 22 mm. As described, grayscale images at 450 nm were then analyzed using the software MATLAB R2017a. The pellets were segmented using the watershed algorithm and multiple images were sorted using the sort_nat function version 1.4 developed by Douglas M. Schwarz. The intensity of the PTO Pellets at 450 nm was transformed into a mean value.

The trend of data was similar between single pixel and entire area analysis (Figure S11). The amount of pixels per sample was ~160 for single pixel analysis and for entire area analysis the amount of pixels was ~16000. Standard deviation of intensity within the image was 11.6 ± 1.9 per image (mean ± st.dev., n=70) for single pixel analysis and 13.9 ± 1.3 per image (mean ± st.dev., n=70) for entire area analysis. The clearest difference between the data treatments was the mean values, which were consequently ~10 values lower for entire area analysis. Mean value of entire data was 196.0 ± 10.9 for single pixel analysis and 184.4 ± 11.0 for entire area (mean ± st.dev., n=70).

Since data trend was so similar between the two data treatments, it was decided to use the single pixel analysis for data treatment, since this was the simplest form. Example of data treatment of images can be found in Table S3.

**
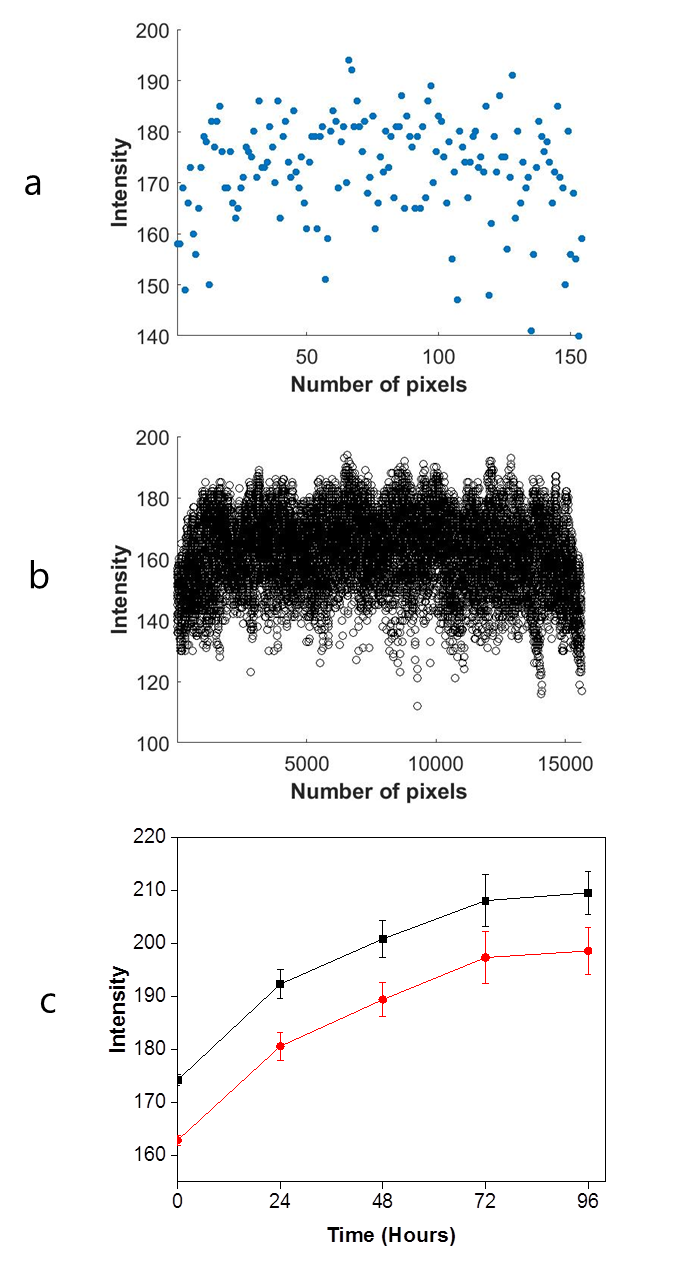
**

**Figure S11.** Comparison between data treatments for image analysis. Example of intensity data for one of the images for: (a) single pixel data treatment and (b) entire area data treatment. Image used was of 17.7 wt% PTO Pellets treated with H_2_O_2_ vapor of 1.20∙10^-7^ atm for 48 hours. Examples of image treatment are found in Table S2. (c): Mean intensity of 17.7 wt% PTO Pellets 1000 treated with H_2_O_2_ vapor of 1.20∙10^-7^ atm for 0-96 hours analyzed with single pixel method (■) or entire area (●) (mean ± st.dev., n=7) (c).

**Table S3.** Data treatment of image of 17.7 wt% PTO Pellets treated with H_2_O_2_ vapor of 1.20∙10^-7^ atm for 48 hours. Comparison of single pixel and entire area data treatment.

| Original picture (.png) | 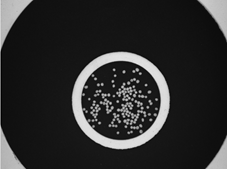 | |
| --- | --- | --- |
| Data area determination  (Removal of white ring) | 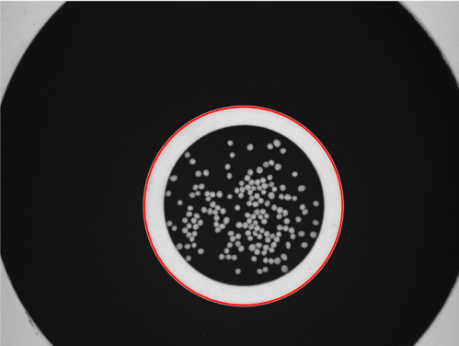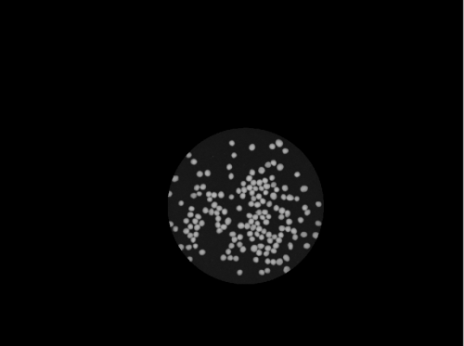 | |
| Imbinarized and cropped image  Data area = white  Non-data = black  (Removal of white ring) | 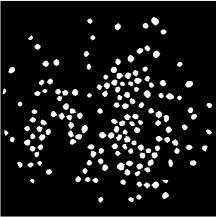 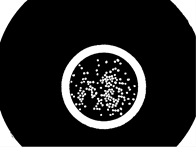 | |
| Data treatment | Single pixel | Entire area |
| Data area  White area is included in analysis.  Green area is not included in analysis  Analysis: Intensity of pixel in grayscale image at 450 nm. | 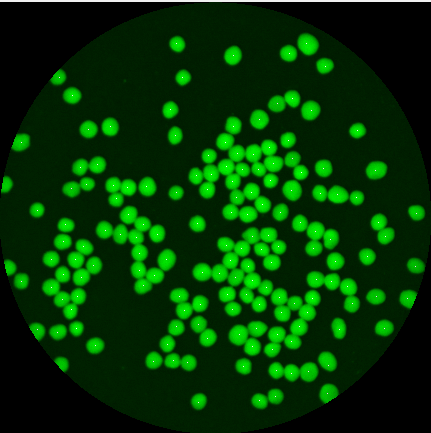 | 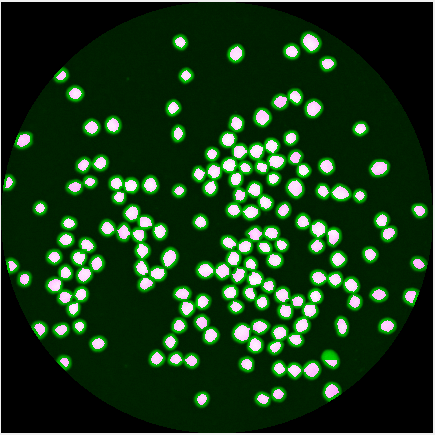 |
